# Ethyl 4-chloro-3,5-dinitro­benzoate

**DOI:** 10.1107/S160053681103978X

**Published:** 2011-10-12

**Authors:** Hao Wu, Min-Hao Xie, Ya-Ling Liu, Yong-Jun He, Pei Zou

**Affiliations:** a Jiangsu Institute of Nuclear Medicine, Wuxi 214063, People’s Republic of China

## Abstract

In the title compound, C_9_H_7_ClN_2_O_6_, the nitro groups and the ester group make dihedral angles of 44.0 (1), 89.6 (1) and 164.1 (1)°, respectively, with the benzene ring. In the crystal, mol­ecules are linked through weak C—H⋯O hydrogen-bonding inter­actions. Mol­ecules are stacked *via* π–π inter­actions about inversion centers, with a centroid–centroid distance of 3.671 (2) Å.

## Related literature

For applications of the title compound as a herbicide and a related structure, see: Liu *et al.* (2010 ▶).
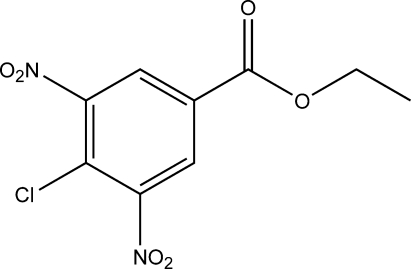


## Experimental

### 

#### Crystal data

C_9_H_7_ClN_2_O_6_
*M* *_r_* = 274.62
Monoclinic, 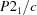

*a* = 7.744 (2) Å
*b* = 21.389 (6) Å
*c* = 7.241 (2) Å
β = 110.504 (4)°
*V* = 1123.3 (5) Å^3^
*Z* = 4
Mo *K*α radiation
μ = 0.36 mm^−1^
*T* = 133 K
0.30 × 0.20 × 0.10 mm

#### Data collection

Rigaku SPIDER diffractometer
Absorption correction: multi-scan (*ABSCOR*; Higashi, 1995 ▶) *T*_min_ = 0.899, *T*_max_ = 0.965
8777 measured reflections
2549 independent reflections
1939 reflections with *I* > 2σ(*I*)
*R*_int_ = 0.034

#### Refinement

*R*[*F* ^2^ > 2σ(*F* ^2^)] = 0.041
*wR*(*F* ^2^) = 0.098
*S* = 1.00
2549 reflections
164 parameters
H-atom parameters constrained
Δρ_max_ = 0.51 e Å^−3^
Δρ_min_ = −0.32 e Å^−3^

### 

Data collection: *RAPID-AUTO* (Rigaku, 2004 ▶); cell refinement: *RAPID-AUTO*; data reduction: *RAPID-AUTO*; program(s) used to solve structure: *SHELXS97* (Sheldrick, 2008 ▶); program(s) used to refine structure: *SHELXL97* (Sheldrick, 2008 ▶); molecular graphics: *SHELXTL* (Sheldrick, 2008 ▶); software used to prepare material for publication: *SHELXTL*.

## Supplementary Material

Crystal structure: contains datablock(s) I, global. DOI: 10.1107/S160053681103978X/pv2450sup1.cif

Structure factors: contains datablock(s) I. DOI: 10.1107/S160053681103978X/pv2450Isup2.hkl

Supplementary material file. DOI: 10.1107/S160053681103978X/pv2450Isup3.cml

Additional supplementary materials:  crystallographic information; 3D view; checkCIF report

## Figures and Tables

**Table 1:** Hydrogen-bond geometry (Å, °)

*D*—H⋯*A*	*D*—H	H⋯*A*	*D*⋯*A*	*D*—H⋯*A*
C5—H5⋯O2^i^	0.95	2.32	3.157 (3)	147

Symmetry code: (i) 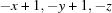.

**Fig. 1.**
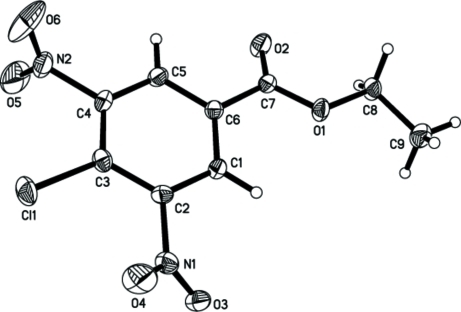
A view of the title compound with the atomic numbering scheme. Displacement ellipsoids were drawn at the 50% probability level.

**Fig. 2.**
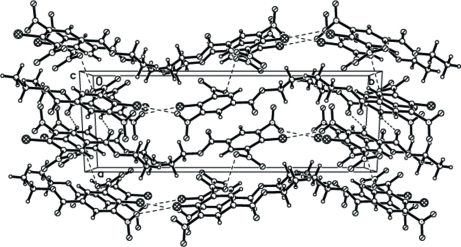
Part of the packing of the title compound, viewed down the c direction; dashed lines indicate hydrogen bonds.

## supplementary crystallographic information

### Comment

The title compound is useful as a herbicide (Liu *et al.*, 2010). In the title molecule (Fig. 1), two nitro groups (O3/N1/O4 and O5/N2/O6) attached at C2 and C4 and the ester group (O1/C7/O2) attached at C6 form dihedral angles of 44.0 (1), 89.6 (1) and 164.1 (1)°, respectively, with the mean plane of the benzene ring (C1–C6). In the cyrstal structure, the molecules are linked through weak C—H···O hydrogen bonding interactions. The molecules are stacked *via*π-π interactions, about inversion centers with the ring centroid-centroid distance of 3.671 (2) Å.

### Experimental

A sample of commercial ethyl 4-chloro-3,5-dinitrobenzoate (Aldrich) was crystalized by slow evaporation of a solution in methanol yielding colorless chunky crystals after several days.

### Refinement

H atoms were placed in calculated positions with C—H = 0.99, 0.98 or 0.95 Å for methylene, methyl or aryl type H-atoms, respectively, and were refined in a riding mode with *U*_iso_(H) = 1.2 or 1.5*U*_eq_(C).

### Crystal data

**Table:**

C_9_H_7_ClN_2_O_6_	*F*(000) = 560
*M**_r_* = 274.62	*D*_x_ = 1.624 Mg m^−^^3^
Monoclinic, *P*2_1_/*c*	Melting point: 357(2) K
Hall symbol: -P 2ybc	Mo *K*α radiation, λ = 0.71073 Å
*a* = 7.744 (2) Å	Cell parameters from 2718 reflections
*b* = 21.389 (6) Å	θ = 3.2–27.5°
*c* = 7.241 (2) Å	µ = 0.36 mm^−^^1^
β = 110.504 (4)°	*T* = 133 K
*V* = 1123.3 (5) Å^3^	Block, colorless
*Z* = 4	0.30 × 0.20 × 0.10 mm

### Data collection

**Table:**

Rigaku SPIDER diffractometer	2549 independent reflections
Radiation source: Rotating Anode	1939 reflections with *I* > 2σ(*I*)
graphite	*R*_int_ = 0.034
ω scans	θ_max_ = 27.5°, θ_min_ = 3.8°
Absorption correction: multi-scan (*ABSCOR*; Higashi, 1995)	*h* = −8→10
*T*_min_ = 0.899, *T*_max_ = 0.965	*k* = −27→27
8777 measured reflections	*l* = −9→9

### Refinement

**Table:**

Refinement on *F*^2^	Primary atom site location: structure-invariant direct methods
Least-squares matrix: full	Secondary atom site location: difference Fourier map
*R*[*F*^2^ > 2σ(*F*^2^)] = 0.041	Hydrogen site location: inferred from neighbouring sites
*wR*(*F*^2^) = 0.098	H-atom parameters constrained
*S* = 1.00	*w* = 1/[σ^2^(*F*_o_^2^) + (0.0496*P*)^2^ + 0.269*P*] where *P* = (*F*_o_^2^ + 2*F*_c_^2^)/3
2549 reflections	(Δ/σ)_max_ < 0.001
164 parameters	Δρ_max_ = 0.51 e Å^−^^3^
0 restraints	Δρ_min_ = −0.32 e Å^−^^3^

### Special details

**Table:**

Geometry. All e.s.d.'s (except the e.s.d. in the dihedral angle between two l.s. planes) are estimated using the full covariance matrix. The cell e.s.d.'s are taken into account individually in the estimation of e.s.d.'s in distances, angles and torsion angles; correlations between e.s.d.'s in cell parameters are only used when they are defined by crystal symmetry. An approximate (isotropic) treatment of cell e.s.d.'s is used for estimating e.s.d.'s involving l.s. planes.
Refinement. Refinement of *F*^2^ against ALL reflections. The weighted *R*-factor *wR* and goodness of fit *S* are based on *F*^2^, conventional *R*-factors *R* are based on *F*, with *F* set to zero for negative *F*^2^. The threshold expression of *F*^2^ > σ(*F*^2^) is used only for calculating *R*-factors(gt) *etc*. and is not relevant to the choice of reflections for refinement. *R*-factors based on *F*^2^ are statistically about twice as large as those based on *F*, and *R*- factors based on ALL data will be even larger.

### Fractional atomic coordinates and isotropic or equivalent isotropic displacement parameters (Å^2^)

**Table:**

	*x*	*y*	*z*	*U*_iso_*/*U*_eq_	
Cl1	0.66615 (7)	0.68101 (2)	0.67393 (8)	0.02902 (15)	
O1	0.82698 (18)	0.39122 (6)	0.43011 (19)	0.0229 (3)	
O2	0.6015 (2)	0.41837 (6)	0.14870 (19)	0.0283 (3)	
O3	0.82102 (19)	0.51915 (7)	1.00581 (19)	0.0269 (3)	
O4	0.9456 (2)	0.60842 (7)	0.9835 (2)	0.0402 (4)	
O5	0.3393 (3)	0.65336 (10)	0.2213 (3)	0.0610 (6)	
O6	0.5882 (3)	0.68236 (8)	0.1760 (3)	0.0562 (6)	
N1	0.8513 (2)	0.56285 (8)	0.9112 (2)	0.0237 (4)	
N2	0.5021 (3)	0.64949 (8)	0.2496 (2)	0.0286 (4)	
C1	0.7812 (2)	0.49987 (8)	0.6145 (3)	0.0172 (4)	
H1	0.8417	0.4660	0.6966	0.021*	
C2	0.7714 (2)	0.55765 (8)	0.6956 (3)	0.0177 (4)	
C3	0.6846 (2)	0.60877 (8)	0.5805 (3)	0.0189 (4)	
C4	0.6034 (2)	0.59805 (8)	0.3785 (3)	0.0194 (4)	
C5	0.6100 (3)	0.54110 (8)	0.2923 (3)	0.0194 (4)	
H5	0.5530	0.5357	0.1539	0.023*	
C6	0.7015 (2)	0.49173 (8)	0.4111 (3)	0.0166 (4)	
C7	0.7039 (2)	0.43024 (8)	0.3140 (3)	0.0178 (4)	
C8	0.8358 (3)	0.32869 (8)	0.3507 (3)	0.0232 (4)	
H8A	0.9010	0.3303	0.2551	0.028*	
H8B	0.7101	0.3121	0.2829	0.028*	
C9	0.9385 (3)	0.28811 (10)	0.5225 (3)	0.0317 (5)	
H9A	1.0599	0.3064	0.5927	0.048*	
H9B	0.9540	0.2463	0.4752	0.048*	
H9C	0.8689	0.2851	0.6121	0.048*	

### Atomic displacement parameters (Å^2^)

**Table:**

	*U*^11^	*U*^22^	*U*^33^	*U*^12^	*U*^13^	*U*^23^
Cl1	0.0369 (3)	0.0164 (2)	0.0335 (3)	0.0007 (2)	0.0121 (2)	−0.00696 (19)
O1	0.0279 (8)	0.0157 (6)	0.0196 (7)	0.0058 (5)	0.0016 (6)	−0.0021 (5)
O2	0.0401 (9)	0.0187 (7)	0.0173 (7)	0.0037 (6)	−0.0012 (6)	−0.0029 (5)
O3	0.0299 (8)	0.0321 (8)	0.0197 (7)	0.0041 (6)	0.0102 (6)	0.0041 (6)
O4	0.0530 (10)	0.0306 (9)	0.0267 (8)	−0.0122 (7)	0.0010 (7)	−0.0100 (6)
O5	0.0495 (12)	0.0778 (15)	0.0620 (13)	0.0438 (10)	0.0275 (10)	0.0343 (11)
O6	0.0571 (12)	0.0344 (10)	0.0572 (12)	−0.0177 (8)	−0.0049 (9)	0.0259 (9)
N1	0.0249 (9)	0.0255 (9)	0.0190 (8)	0.0027 (7)	0.0058 (7)	−0.0032 (7)
N2	0.0392 (11)	0.0175 (9)	0.0226 (9)	0.0054 (8)	0.0027 (8)	0.0015 (7)
C1	0.0177 (9)	0.0149 (8)	0.0176 (9)	0.0010 (7)	0.0046 (7)	0.0016 (7)
C2	0.0189 (10)	0.0195 (9)	0.0140 (9)	−0.0019 (7)	0.0048 (7)	−0.0009 (7)
C3	0.0194 (10)	0.0146 (9)	0.0233 (9)	−0.0019 (7)	0.0081 (8)	−0.0029 (7)
C4	0.0211 (10)	0.0151 (9)	0.0211 (9)	0.0026 (7)	0.0063 (8)	0.0042 (7)
C5	0.0224 (10)	0.0180 (9)	0.0164 (9)	0.0001 (7)	0.0051 (8)	0.0015 (7)
C6	0.0180 (9)	0.0146 (8)	0.0172 (8)	−0.0006 (7)	0.0062 (7)	−0.0001 (7)
C7	0.0210 (10)	0.0153 (9)	0.0179 (9)	0.0010 (7)	0.0077 (8)	0.0017 (7)
C8	0.0300 (11)	0.0140 (9)	0.0230 (10)	0.0032 (8)	0.0062 (8)	−0.0029 (7)
C9	0.0426 (13)	0.0225 (11)	0.0285 (11)	0.0077 (9)	0.0104 (10)	0.0031 (8)

### Geometric parameters (Å, °)

**Table:**

Cl1—C3	1.7125 (19)	C2—C3	1.396 (3)
O1—C7	1.323 (2)	C3—C4	1.394 (3)
O1—C8	1.467 (2)	C4—C5	1.378 (3)
O2—C7	1.209 (2)	C5—C6	1.389 (2)
O3—N1	1.229 (2)	C5—H5	0.9500
O4—N1	1.220 (2)	C6—C7	1.495 (2)
O5—N2	1.207 (2)	C8—C9	1.497 (3)
O6—N2	1.213 (2)	C8—H8A	0.9900
N1—C2	1.468 (2)	C8—H8B	0.9900
N2—C4	1.478 (2)	C9—H9A	0.9800
C1—C2	1.382 (2)	C9—H9B	0.9800
C1—C6	1.394 (2)	C9—H9C	0.9800
C1—H1	0.9500		
			
C7—O1—C8	116.64 (14)	C4—C5—H5	120.6
O4—N1—O3	124.79 (17)	C6—C5—H5	120.6
O4—N1—C2	118.86 (16)	C5—C6—C1	120.02 (16)
O3—N1—C2	116.32 (16)	C5—C6—C7	117.72 (16)
O5—N2—O6	126.00 (19)	C1—C6—C7	122.22 (16)
O5—N2—C4	116.76 (18)	O2—C7—O1	124.99 (17)
O6—N2—C4	117.18 (18)	O2—C7—C6	122.60 (16)
C2—C1—C6	119.39 (16)	O1—C7—C6	112.40 (15)
C2—C1—H1	120.3	O1—C8—C9	106.70 (16)
C6—C1—H1	120.3	O1—C8—H8A	110.4
C1—C2—C3	122.25 (17)	C9—C8—H8A	110.4
C1—C2—N1	117.00 (16)	O1—C8—H8B	110.4
C3—C2—N1	120.72 (16)	C9—C8—H8B	110.4
C4—C3—C2	116.31 (16)	H8A—C8—H8B	108.6
C4—C3—Cl1	119.57 (14)	C8—C9—H9A	109.5
C2—C3—Cl1	124.07 (15)	C8—C9—H9B	109.5
C5—C4—C3	123.11 (17)	H9A—C9—H9B	109.5
C5—C4—N2	117.89 (17)	C8—C9—H9C	109.5
C3—C4—N2	118.99 (16)	H9A—C9—H9C	109.5
C4—C5—C6	118.89 (17)	H9B—C9—H9C	109.5
			
C6—C1—C2—C3	−0.3 (3)	O5—N2—C4—C3	−90.7 (2)
C6—C1—C2—N1	177.67 (16)	O6—N2—C4—C3	92.1 (2)
O4—N1—C2—C1	136.26 (18)	C3—C4—C5—C6	0.1 (3)
O3—N1—C2—C1	−41.8 (2)	N2—C4—C5—C6	−178.79 (17)
O4—N1—C2—C3	−45.8 (3)	C4—C5—C6—C1	1.4 (3)
O3—N1—C2—C3	136.14 (17)	C4—C5—C6—C7	179.03 (16)
C1—C2—C3—C4	1.6 (3)	C2—C1—C6—C5	−1.3 (3)
N1—C2—C3—C4	−176.24 (16)	C2—C1—C6—C7	−178.82 (16)
C1—C2—C3—Cl1	179.17 (14)	C8—O1—C7—O2	−1.0 (3)
N1—C2—C3—Cl1	1.3 (3)	C8—O1—C7—C6	177.99 (15)
C2—C3—C4—C5	−1.5 (3)	C5—C6—C7—O2	−15.2 (3)
Cl1—C3—C4—C5	−179.18 (15)	C1—C6—C7—O2	162.35 (18)
C2—C3—C4—N2	177.30 (17)	C5—C6—C7—O1	165.76 (16)
Cl1—C3—C4—N2	−0.4 (2)	C1—C6—C7—O1	−16.6 (2)
O5—N2—C4—C5	88.2 (2)	C7—O1—C8—C9	−163.27 (17)
O6—N2—C4—C5	−89.0 (2)		

### Hydrogen-bond geometry (Å, °)

**Table:**

*D*—H···*A*	*D*—H	H···*A*	*D*···*A*	*D*—H···*A*
C5—H5···O2^i^	0.95	2.32	3.157 (3)	147

Symmetry codes: (i) −*x*+1, −*y*+1, −*z*.
